# Prescription practice of anti-tuberculosis drugs in Yunnan, China: A clinical audit

**DOI:** 10.1371/journal.pone.0187076

**Published:** 2017-10-31

**Authors:** Lin Xu, Jinou Chen, Anh L. Innes, Ling Li, Chen-Yuan Chiang

**Affiliations:** 1 Tuberculosis Division, Yunnan Center of Disease Control, Yunnan, China; 2 FHI 360 Asia Pacific Regional Office, Bangkok, Thailand; 3 FHI 360 China (Kunming), Yunnan, China; 4 International Union Against Tuberculosis and Lung Disease, Paris, France; 5 Division of Pulmonary Medicine, Department of Internal Medicine, Wan Fang Hospital, Taipei Medical University, Taipei, Taiwan; 6 Division of Pulmonary Medicine, Department of Internal Medicine, School of Medicine, College of Medicine, Taipei Medical University, Taipei, Taiwan; Indian Institute of Technology Delhi, INDIA

## Abstract

**Objectives:**

China has a high burden of drug-resistant tuberculosis (TB). As irrational use and inadequate dosing of anti-TB drugs may contribute to the epidemic of drug-resistant TB, we assessed the drug types and dosages prescribed in the treatment of TB cases in a representative sample of health care facilities in Yunnan.

**Methods:**

We applied multistage cluster sampling using probability proportion to size to select 28 counties in Yunnan. Consecutive pulmonary TB patients were enrolled from either the TB centers of Yunnan Center of Disease Control or designated TB hospitals. Outcomes of interest included the regimen used in the treatment of new and retreatment TB patients; and the proportion of patients treated with adequate dosing of anti-TB drugs. Furthermore, we assess whether there has been reduction in the use of fluoroquinolone and second line injectables in Tuberculosis Clinical Centre (TCC) after the training activity in late 2012.

**Results:**

Of 2390 TB patients enrolled, 582 (24.4%) were prescribed second line anti-TB drugs (18.0% in new cases and 60.9% in retreatment cases); 363(15.2%) prescribed a fluoroquinolone. General hospitals (adjusted odds ratio (adjOR) 1.97, 95% confidence interval (CI) 1.47–2.66), retreatment TB cases (adjOR 4.75, 95% CI 3.59–6.27), smear positive cases (adjOR 1.69, 95% CI 1.22–2.33), and extrapulmonary TB (adjOR 2.59, 95% CI 1.66–4.03) were significantly associated with the use of fluoroquinolones. The proportion of patients treated with fluoroquinolones decreased from 41.4% before 2013 to 13.5% after 2013 (adjOR 0.19, 95% CI 0.12–0.28) in TCC. The proportion of patients with correct, under and over dosages of isoniazid was 88.2%, 1.5%, and 10.4%, respectively; of rifampicin was 50.2%, 46.8%, and 2.9%; of pyrazinamide was 67.6%, 31.7% and 0.7%; and of ethambutol was 41.4%, 57.5%, and 1.0%.

**Conclusions:**

The prescribing practice of anti-TB drugs was not standardized, findings with significant programmatic implication.

## Introduction

Tuberculosis (TB) remains a major public health problem in China, although there has been a remarkable decrease in the country’s TB burden in past decades [1]. China also has a high burden of drug-resistant TB. The recent national anti-TB drug resistance survey revealed that the proportion of multidrug-resistant tuberculosis (MDR-TB) among new cases was 5.6% and that among previously treated cases 25.6% [2].

TB control depends on early diagnosis and effective treatment that render infectious TB cases non-infectious. The standard first-line anti- TB regimen recommended by Chinese authorities for new TB patients consists of isoniazid (INH, H), rifampicin (RMP, R), pyrazinamide (PZA, Z) and ethambutol (EMB, E) daily for 2 months, followed by HR daily for 4 months, and that for previously treated TB patients H, R, Z, E and streptomycin (SM, S) daily for 2 months, followed by HER daily for 6 months. The alternative regimens are intermittent therapy administering medicines every other day in higher dosages throughout the whole treatment course. The recommended regimens for the treatment of MDR-TB were in line with the recommendations of the World Health Organization [3].

It has been reported that second line anti-TB drugs were widely available in health care facilities at provincial, prefecture, and county levels in China [4], and that a high proportion of TB patients in China were prescribed anti-TB treatment regimens that were not consistent with national recommendations [5]. Review of prescription practices has been indicated as an essential tool for measuring the quality of TB services [6]. Prescription of adequate regimens and appropriate dosages of anti-TB medications are critical, as inadequate regimen and inappropriate dosing may result in unfavourable treatment outcomes and acquired resistance to anti-TB medicines. Prescription practice and dosing of anti-TB medications have been evaluated in some settings [7–10].

Yunnan province has an annual notification rate of TB of 54.4 per 100,000 population in 2015. Starting in 2012, Yunnan has implemented the Control and Prevention of TB (CAP-TB) project, funded by the United States Agency for International Development (USAID). The CAP-TB project is led by FHI 360, partnered with the International Union Against Tuberculosis and Lung Disease, and it has included prevention and management of drug-resistant TB as part of its priority activities. In the project’s early stage, it was noted during field visits that second line drugs were frequently used in the Yunnan Center of Disease Control’s (CDC) Tuberculosis Clinical Centre (TCC). Rational use of fluoroquinolones and second line injectables in the treatment of TB was highlighted in a training activity in TCC in late 2012 and thereafter repeatedly emphasized during regular field visits. As the prescription practice of anti-TB drugs has not been assessed in Yunnan, we conducted a clinical audit on the type and dosages of drugs used in a selected sample of health care facilities in Yunnan and assessed change of prescription practice in TCC. We report the findings of this clinical audit.

## Materials and methods

TB services (diagnosis and treatment) in Yunnan are mainly provided at TB centers of Yunnan CDC at provincial, prefecture, and county levels. China has established a national policy in 2008 that TB services should be gradually switched from CDC’s TB centers to general hospitals that are selected as designated TB hospitals. In Yunnan, the transition of TB services from CDC’s TB centers to general hospitals has been initiated but is not yet complete. By the end of 2016, 71 (55%) of the 129 counties in Yunnan has established designated TB hospitals.

Yunnan starting implementing programmatic management of drug-resistant TB (PMDT) in 2012. Anti-TB drug susceptibility testing (DST) is mainly performed at the mycobacteriology laboratory of Yunnan Provincial CDC, which has passed the DST proficiency testing by the national mycobacteriology reference laboratory in Beijing since 2008. MDR-TB patients in Yunnan are mainly managed at the provincial level in the Yunnan TCC, and will eventually be scaled up through decentralization to the designated TB hospitals at prefecture level. The objective of this study is to investigate the quality of prescription practice in the management of new and retreatment TB before scaling–up PMDT. Prescription practice in the management of MDR-TB is not the focus of this study.

We planned to assess 1) whether there was irrational use of second line anti-TB drugs in the treatment of new and retreatment TB patients who were not laboratory-diagnosed with MDR-TB in Yunnan, and 2) whether the use of fluoroquinolones and second line injectables decreased after the training activity in late 2012 for new and retreatment TB patients who were not laboratory-diagnosed with MDR-TB in Yunnan TCC, and 3) whether dosing of anti-TB drugs was in line with national and international recommendations.

Yunnan has 16 prefectures and 129 counties. We applied multistage cluster sampling using probability proportion to size (PPS) to select 28 counties for this study, aiming to enroll 1500 patients. The number of counties selected in each prefecture was allocated according to the number of all forms of TB patients notified in 2014. Cases were enrolled either in the CDC TB centers or designated TB hospitals depending on the status of transition of TB services from the CDC TB centers to designated TB hospitals. In counties where both the CDC TB centers and designated TB hospitals were providing TB services, cases were enrolled at both sites. Consecutive new and retreatment pulmonary TB patients who were admitted since January 2015 were enrolled; those who were treated at outpatient department but not admitted were not enrolled in this study because their medical records were usually kept with patients and not available for review.

To assess whether there has been reduction in the use of fluoroquinolone and second line injectables in Yunnan TCC after the training activity in late 2012, we enrolled consecutive new and retreatment pulmonary TB patients who were admitted in TCC from September 2011 –June 2014 aiming to enroll at least 400 patients.

### Data collection

A standardized questionnaire was developed for data collection, which included sex, age at diagnosis of TB, type of case (new, retreatment including relapse, treatment after loss-to-follow-up, and treatment after failure), smear for acid-fast bacilli, culture for *M*. *tuberculosis*, drug susceptibility testing, body weight at baseline, date of treatment initiation, type of clinician who initiated treatment (junior vs senior), status of health insurance of patients (with or without), frequency of drugs administered (daily or intermittently), drugs used including INH, RMP, PZA, EMB, SM, rifapentine, rifabutin, 4-drug fixed dose combination (FDC) of HRZE (FDC-HRZE, INH 75mg, RMP 150mg, EMB 400mg, PZA 400 mg), 2-drug FDC of HR (FDC-HR-1, INH 75mg, RMP 300mg; and FDC-HR-2, INH 75mg, RMP 150mg), 2-drug FDC of H and PAS (FDC-HP (dipasic), INH 47.3 mg, PAS 52.7 mg) [11–13], blister pack of HRZE (BP-HRZE, INH 600mg, RMP 600mg, EMB 1250mg, PZA 2000 mg), blister pack of HER (BP-HER, INH 600mg, RMP 600mg, EMB 1250mg), blister pack of HR (BP-HR, INH 600mg, RMP 600mg), ofloxacin, levofloxacin, moxifloxacin, gatifloxacin, kanamycin, amikacin, capreomycin, protionamide, cycloserine, PAS, amoxicillin/clavulanate, clarithromycin, linezolid, clofazimine, as well as frequency and dosage of each drug prescribed. We classify INH, RMP, PZA, EMB, SM, rifapentine, rifabutin, FDC-HRZE, FDC-HR-1, FDC-HR-2, dipasic, BP-HRZE, BP-HER, and BP-HR as first line drugs.

Data collection was done by trained health workers of the Yunnan CDC who were not health care providers of the health care facilities included in this study. Medical charts of patients were obtained from health care facilities, and reviewed for data collection.

### Data management and analysis

The complete data set was entered using EpiData Entry 3.1 (EpiData Association, Odense, Denmark). R software (R Core Team 2016; http://www.R-project.org) was used for statistical analysis.

Outcomes of interest included the regimen used in the treatment of new and retreatment TB patients; the proportion of new and retreatment pulmonary TB patients treated with second line anti-TB drugs, especially fluoroquinolones and second line injectables; and the proportion of patients treated with adequate dosing of anti-TB drugs.

Adequacy of dosing of anti-TB drugs was assessed by comparing dosage prescribed with Chinese NTP guidelines (S1 Table) as well as internationally- recommend dosages in mg/kg body weight: 5 (4–6) mg/kg for INH, 10 (8–12) mg/kg for RMP, 15 (15–20) mg/kg for EMB, 25(20–30) mg/kg for PZA, and 15 (12–18) mg/kg for SM [14].

Categorical variables were analyzed using Pearson’s χ2 test. To assess factors associated with the use of fluoroquinolone and second line injectables in the treatment of TB, we constructed saturated logistic regression models including all potential variables in the models, and applied a backwards elimination method to determine the models with the best fit. Three methods were used to handle missing values: 1) dummy variables created for missing values, 2) multiple imputation, and 3) data included were only those without missing values. To assess factors associated with adequate dosing, multinomial logistic regression models were constructed with the following categories: adequate dosage, lower-than-recommended dosage and higher-than-recommended dosage. *P* < 0.05 was considered statistically significant.

### Ethics considerations

Ethics clearance was obtained from the Institution Review Board of Yunnan CDC (approval number 2015–01), the Office of International Research Ethics and Protection of Human Subjects Committee at FHI 360 (IRBNet Project 745967–1), and the Ethics Advisory Group of The Union (EAG number 39/15).

## Results

Medical charts were reviewed from 798 TB patients treated at the provincial level, in Yunnan TCC, and 1681 at county hospitals. Of the 798 patients treated at Yunnan TCC, 89 patients were excluded because of MDR-TB (n = 83) and uncertainty of the diagnosis of TB (n = 6). Table 1 shows characteristics of the 2390 TB patients who were included in this study. Of the 2390 patients, 1347 (56.4%) were treated at TCC and the CDC TB centers, and 1043 (43.6%) at general hospitals designated as TB hospitals; 1941 (81.2%) were new TB patients, 389 (16.3%) were smear positive.

**Table 1:** Demographics of the study population, by county health care facilities (CDC’s TB center or designated TB hospital) and Tuberculosis Clinical Center (TCC).

	Total		County health care facilities		TCC	
	Number	%	Number	%	Number	%
Total	2390	100.0	1681	100.0	709	100.0
Sex						
Male	1573	65.8	1118	66.5	455	64.2
Female	812	34.0	559	33.3	253	35.7
Unknown	5	0.2	4	0.2	1	0.1
Age groups (years)						
<25	415	17.4	318	18.9	97	13.7
25–44	848	35.5	540	32.1	308	43.4
44–65	799	33.4	561	33.4	238	33.6
65 or more	318	13.3	258	15.3	60	8.5
Unknown	10	0.4	4	0.2	6	0.8
Type of facility						
CDC’s TB centers	1347	56.4	638	38.0	709	100.0
General hospitals	1043	43.6	1043	62.0	0	0.0
Type of case						
New	1941	81.2	1428	84.9	513	72.4
Retreatment	317	13.3	156	9.3	161	22.7
Unknown	132	5.5	97	5.8	35	4.9
Sputum smear for AFB						
Negative	1728	72.3	1360	80.9	368	51.9
Positive	389	16.3	124	7.4	265	37.4
Not done/unknown	273	11.4	197	11.7	76	10.7
Body weight (kg)						
<50	904	37.8	639	38.0	265	37.4
50 or more	1417	59.3	983	58.5	434	61.2
Unknown	69	2.9	59	3.5	10	1.4
Health insurance						
No	567	23.7	95	5.7	472	66.6
Yes	1780	74.5	1543	91.8	237	33.4
Unknown	43	1.8	43	2.6	0	0.0
Type of clinician						
Junior	925	38.7	490	29.1	435	61.4
Senior	1372	57.4	1129	67.2	243	34.3
Unknown	93	3.9	62	3.7	31	4.4
Site of disease						
Extra-pulmonary TB	156	6.5	0	0.0	156	22.0
Pulmonary TB	2234	93.5	1681	100.0	553	78.0

Note: CDC, Centers of Disease Control; AFB, acid-fast bacilli

**Fig 1:**
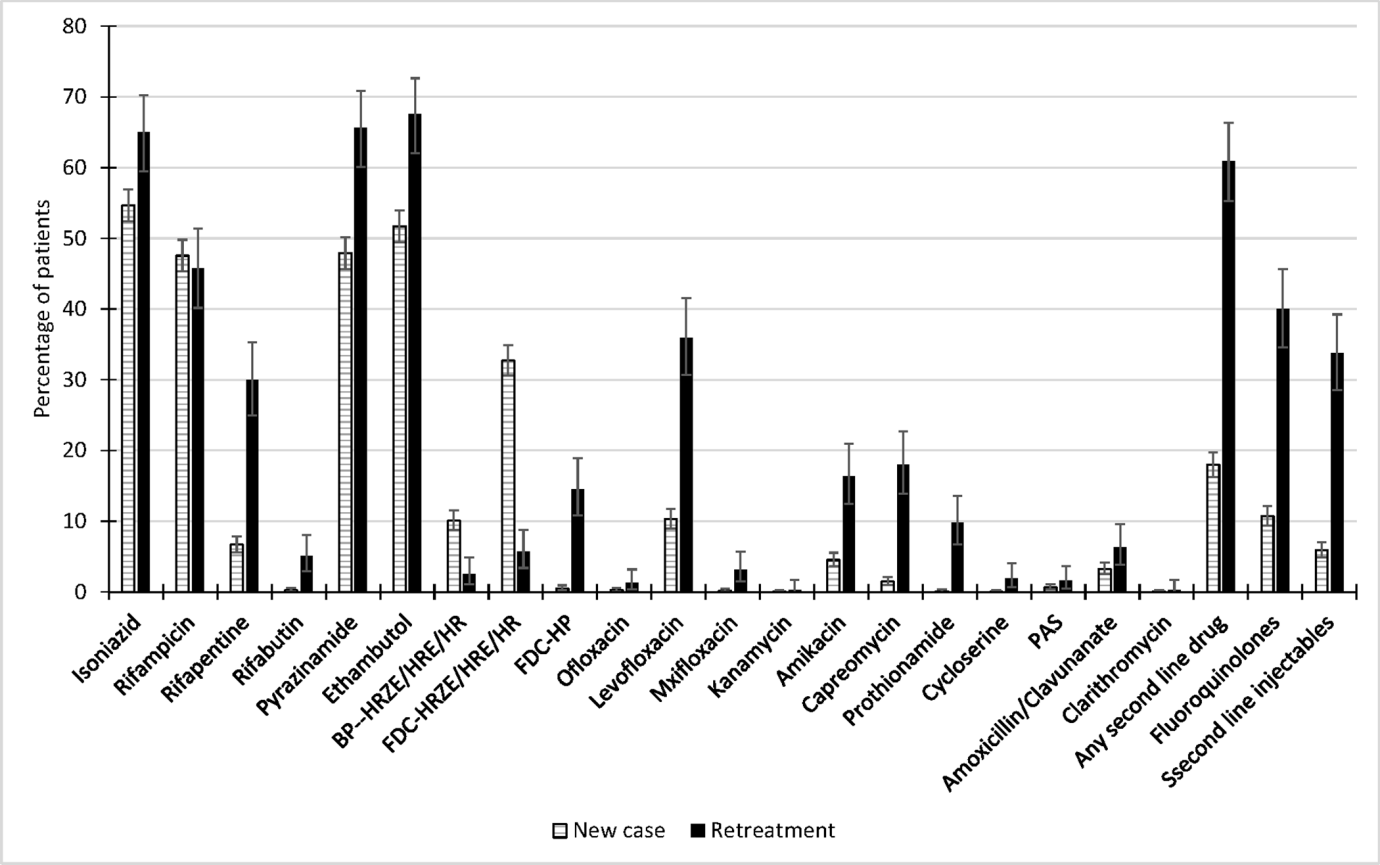
Proportion of tuberculosis patients treated with each type of anti-tuberculosis drugs, with 95% confidence interval, by new and previously treated cases. (fixed dose combination, FDC; isoniazid, H; rifampicin, R; pyrazinamide, Z; ethambutol, E; PAS, P).

Of the 2390 patients, 1337 (55.9%) were prescribed INH, 1134 (47.4%) RMP, 233 (9.7%) rifapentine, 21 (0.9%) rifabutin, 1203 (50.3%) PZA, 1290 (54.0%) EMB, 6 (0.3%) SM, 212 (8.9%) blister packs of HRZE, HER, or HR, 679 (28.4%) FDC- HRZE or HR, 63(2.6%) dipasic, 582 (24.4%) at least one second line drugs, 363(15.2%) a fluoroquinolone (341 levofloxacin, 10 ofloxacin, 12 moxifloxacin), 228 (9.5%) a second line injectable agent (145 amikacin, 2 kanamycin, 81 capreomycin), 33 (1.4%) prothionamide, 8(0.3%) cycloserine, 19 (0.8%) PAS, 94 (3.9%) amoxicillin/clavulanate, and 2 (0.1%) clarithromycin. Fig 1 shows the proportions of patients prescribed first and second line drugs, with 95% confidence interval, by type of case.

A total of 189 regimens were prescribed in the treatment of new and retreatment TB patients, shown in Table 2. Of the 2390 patients, 1808(75.6%) were treated with regimens of first line drugs, 512 (21.4%) with regimens of both first line and second line drugs, and 70 (2.9%) with regimens of second line without first line drugs. 18.0% of new TB cases and 60.9% of retreatment TB cases were prescribed at least one second line anti-TB drug. Among 1941 new TB patients, 1452 (74.8%) were treated with HRZE. Among 317 retreatment patients, 6 (1.9%) were treated with HRZES, 34 (10.7%) were treated with HRZE plus one second line injectable agent.

**Table 2:** Regimens used in the treatment of new and retreatment tuberculosis cases, Yunnan.

	All		New case		Retreatment		Unknown	
	Number	Col%	Number	Col%	Number	Col%	Number	Col%
Total	2390	100.0	1941	100.0	317	100.0	132	100.0
First line without second line drugs	1808	75.6	1592	82.0	124	39.1	92	69.7
HRZES	6	0.3	0	0.0	6	1.9	0	0.0
HRZE	1632	68.3	1452	74.8	95	30.0	85	64.4
HRES	2	0.1	1	0.1	1	0.3	0	0.0
HRE/HRZ/HZE/RZE	109	4.6	89	4.6	16	5.0	4	3.0
≦two first line drugs	59	2.5	50	2.6	6	1.9	3	2.3
Both first line and second line drugs	512	21.4	312	16.1	179	56.5	21	15.9
Fluoroquinoline plus other drugs	331	13.8	193	9.9	120	37.9	18	13.6
Second line injectables plus HRZE	101	4.2	65	3.3	34	10.7	2	1.5
Second line injectables plus other drugs	123	5.1	48	2.5	72	22.7	3	2.3
Others	107	4.5	57	2.4	48	2.0	2	0.1
Second line without first line drugs	70	2.9	37	1.9	14	4.4	19	14.4

Note: H, isoniazid; R, rifampicin; Z, pyrazinamide; E, ethambutol; S, streptomycin.

Table 3 shows the factors associated with the use of fluoroquinolones in the treatment of TB. Clinicians in general hospitals were statistically significantly more likely to use fluoroquinolone as compared with the CDC TB centers (adjusted odds ratio (adjOR) 1.97, 95% confidence interval (CI) 1.47–2.66). Retreatment TB cases (adjOR 4.75, 95% CI 3.59–6.27), smear positive cases (adjOR 1.69, 95% CI 1.22–2.33), and extrapulmonary TB (adjOR 2.59, 95% CI 1.66–4.03) were statistically significantly more likely to be treated with fluoroquinolones as compared with new TB cases, smear negative cases, and pulmonary TB, respectively. Those without health insurance were more likely to be treated with fluoroquinolones than those with health insurance (adjOR 2.52, 95% CI 1.89–3.35). The findings were unchanged in the second multivariate analysis that applied multiple imputation and the third analysis including only data without missing values.

**Table 3:** Factors associated with the use of fluoroquinolone in the treatment of new and retreatment tuberculosis cases, Yunnan.

	Total	Use of fluoroquinolone		Univariate analysis			Multivariate analysis		
		Number	%	Odds ratio	95% CI Low	95% CI High	Adjusted Odds ratio	95% CI Low	95% CI High
Sex									
Male	1573	249	15.8	1.16	0.92	1.48			
Female	812	113	13.9	ref					
Unknown	5	4	80.0	1.55	0.08	10.57			
Age groups (years)									
<25	415	44	10.6	ref					
25–44	848	140	16.5	1.67	1.17	2.42			
45–64	799	135	16.9	1.71	1.20	2.49			
65 or more	318	42	13.2	1.28	0.82	2.02			
Unknown	10	2	20.0	2.11	0.31	8.74			
Facility									
CDCs*	1347	198	14.7	ref			ref		
General hospitals	1043	165	15.8	1.09	0.87	1.36	1.97	1.47	2.66
Type of case									
New	1941	208	10.7	ref			ref		
Retreatment	317	127	40.1	5.57	4.26	7.27	4.75	3.59	6.27
Unknown	132	28	21.2	2.24	1.42	3.44	1.75	1.07	2.77
Sputum smear AFB									
Negative	1728	219	12.7	ref			ref		
Positive	389	87	22.4	1.98	1.50	2.61	1.69	1.22	2.33
Not done/ Unknown	273	57	20.9	1.82	1.31	2.50	1.15	0.80	1.63
Insurance									
No	567	156	27.5	2.92	2.30	3.69	2.52	1.89	3.35
Yes	1780	205	11.5	ref			ref		
Unknown	43	2	4.7	0.37	0.06	1.23	0.35	0.06	1.22
Level of clinicians									
Junior	925	155	16.8	1.26	1.00	1.59			
Senior	1372	189	13.8	ref					
Unknown	93	19	20.4	1.61	0.92	2.67			
Site of disease									
Extrapulmonary	156	48	30.8	2.71	1.87	3.86	2.59	1.66	4.03
Pulmonary TB	2234	315	14.1	ref			ref		

Note: CI, confidence interval; CDC, Center of Disease Control; AFB, acid fast bacilli.

* Including Tuberculosis Clinical Centre.

Table 4 shows factors statistically significantly associated with the use of second line injectable agents in the treatment of TB: clinicians in the CDC TB centers compared to that in general hospitals (adjOR 3.67, 95% CI 2.35–5.90), retreatment TB cases compared to new TB cases (adjOR 7.74, 95% CI 5.50–10.94), smear positive cases compared to smear negative cases (adjOR 1.80, 95% CI 1.25–2.59), those without health insurance compared to those with (adjOR 2.96, 95% CI 2.12–4.14). Junior clinicians compared to senior clinicians (adjOR 1.90, 95% CI 1.37–2.66).

**Table 4:** Factors associated with the use of second line injectable agents in the treatment of new and retreatment tuberculosis cases, Yunnan.

	Total	Use of second line injectable agents		Univariate analysis			Multivariate analysis		
		Number	%	Odds ratio	95% CI Low	95% CI High	Adjusted Odds ratio	95% CI Low	95% CI High
Sex									
Male	1573	153	9.7	1.06	0.79	1.42			
Female	812	75	9.2	ref					
Unknown	5	0	0.0	0.00	-	>100			
Age groups (years)									
<25	415	34	8.2	ref					
25–44	848	102	12.0	1.53	1.03	2.33			
45–64	799	78	9.8	1.21	0.80	1.87			
65 or more	318	12	3.8	0.44	0.22	0.84			
Unknown	10	2	20.0	2.80	0.41	11.73			
Facility									
CDCs*	1347	195	14.5	5.18	3.60	7.69	3.67	2.35	5.90
General hospitals	1043	33	3.2	ref			ref		
Type of case									
New	1941	115	5.9	ref			ref		
Retreatment	317	107	33.8	8.09	6.00	10.92	7.74	5.50	10.94
Unknown	132	6	4.5	0.76	0.29	1.61	0.85	0.31	1.97
Sputum smear AFB									
Negative	1728	102	5.9	ref			ref		
Positive	389	89	22.9	4.73	3.47	6.45	1.80	1.25	2.59
Not done/ Unknown	273	37	13.6	2.50	1.66	3.70	2.74	1.70	4.38
Insurance									
No	567	139	24.5	6.17	4.64	8.24	2.96	2.12	4.14
Yes	1780	89	5.0	ref			ref		
Unknown	43	0	0.0	0.00	0.00	9.89	0.00	0.00	8.44
Level of clinicians									
Junior	925	143	15.5	2.88	2.17	3.84	1.90	1.37	2.66
Senior	1372	82	6.0	ref			ref		
Unknown	93	3	3.2	0.52	0.13	1.44	0.34	0.08	1.08
Site of disease									
Extrapulmonary	156	34	21.8	2.93	1.92	4.36			
Pulmonary	2234	194	8.7	ref					

Note: CI, confidence interval; CDC, Center of Disease Control; AFB, acid fast bacilli.

* Including Tuberculosis Clinical Centre.

Table 5 shows changes in the prescription of fluoroquinolones and second line injectable agents in TCC before January 2013 and after January 2013. The proportion of patients treated with fluoroquinolones decreased from 41.4% before 2013 to 13.5% after 2013 (adjOR 0.19, 95% CI 0.12–0.28); however, there was no statistically significant change in the use of second line injectable agents (adjOR 1.06, 95% CI 0.72–1.56).

**Table 5:** Changes in the prescribing of fluoroquinolone and second line injectable agents before and after 2013 in Tuberculosis Treatment Center, Yunnan Center of Disease Control, Yunnan, China.

	Total	Drug used		Univariate analysis			Multivariate analysis		
		Number	%	Odds ratio	95% CI Low	95% CI High	Adjusted odds ratio*	95% CI Low	95% CI High
Use Fluoroquinolone									
Before 2013	290	120	41.4	Ref			Ref		
2013 and after	379	51	13.5	0.22	0.15	0.32	0.19	0.12	0.28
Use second line injectables									
Before 2013	290	78	26.9	Ref			Ref		
2013 and after	379	112	29.6	1.14	0.81	1.61	1.06	0.72	1.56

* Adjusted for age, sex, type of case, smear, health insurance, level of clinician, and site of disease.

Note: CI, confidence interval.

Of the 1337, 1134, 1203, 1290 patients treated INH, RMP, PZA, and EMB, respectively, 1285 (96.1%), 1085 (95.7%), 1158 (96.3%), and 1243 (96.4%) had information on both body weight and dosages prescribed. Of the 1285 patients treated with INH, 1133 (88.2%) were prescribed INH 300mg per day, 130 (10.1%) more than 300mg, and 19 (1.5%) less than 300mg. Of the 1085 patients treated with RMP, 785 (72.4%) were prescribed RMP 450mg per day, 225 (20.7%) more than 450mg, and 75 (6.9%) less than 450mg. Of the 1158 patients treated with PZA, 783 (67.6%) were prescribed PZA 1500mg per day, 8 (0.7%) more than 1500mg, and 367 (31.7%) less than 1500mg. Of the 1243 patients treated with EMB, 1159 (92.3%) were prescribed EMB 750mg per day, 44 (3.5%) more than 750mg, and 367 (31.7%) less than 750mg. According to guidelines of China NTP, the proportion of patients with correct, under and over dosages of INH was 88.2%, 1.5%, and 10.4%, respectively; and that of RMP 50.2%, 46.8%, and 2.9%, respectively; and that of PZA 67.6%, 31.7% and 0.7%, respectively; and that of EMB 41.4%, 57.5%, and 1.0%, respectively. Table 6 shows the proportion of patients with correct, under and over dosages of INH, RMP, PZA, and EMB according to WHO recommendations, as well as the proportion of patients with correct dosages of each drug by sex, age group, type of case, smear status, body weight, and type of facility. Tables 7–10 shows factors associated with inadequate dosing of INH, RMP, PZA and EMB, respectively.

**Table 6:** Proportion of patients with correct, under, and over dosages of anti-tuberculosis drugs prescribe according to dosage in mg per kg body weight recommended by WHO.

	Isoniazid (H)	Rifampicin (R)	Pyrazinamide (Z)	Ethambutol (E)
	N = 1285	N = 1058	N = 1158	N = 1243
	%			
All cases				
Correct dosage	49.3	73.8	60.4	44.9
Under dosage	1.6	21.1	15.5	50.8
Over dosage	49.2	5.1	24.1	4.3
Correct dosage by sex				
Male	59.8	70.8	66.0	36.2
Female	30.2	79.0	50.2	61.0
Correct dosage by age group (years)				
<25	40.8	78.6	57.0	49.0
25–44	50.0	77.7	61.0	45.9
45–64	54.0	68.4	64.1	41.4
65 or more	46.1	68.6	50.4	45.9
Correct dosage by type of case				
New	50.3	75.1	60.2	44.8
Retreatment	41.5	68.5	59.6	46.9
Correct dosage by smear				
Negative	51.2	73.1	60.9	43.8
Positive	42.0	76.0	62.4	47.4
Correct dosage by weight (kg)				
<40	2.2	42.0	47.5	36.6
40–49	0.2	89.1	32.7	94.8
50 or more	83.9	68.5	78.6	15.3
Correct dosage by Facility				
CDCs	48.8	77.5	66.6	40.0
General hospitals	49.6	71.0	54.8	49.0

Note: missing information on sex, n = 1; age n = 6; type of case n = 67; smear n = 202.

**Table 7:** Factors associated with inadequate dosing of Isoniazid, Yunnan.

	Lower-than-recommended dosage*			Higher-than-recommended dosage*		
	Relative RR	95% CI Low	95% CI High	Relative RR	95% CI Low	95% CI High
Sex						
Male	0.39	0.15	1.00	0.27	0.21	0.36
Female	ref			ref		
Age groups (years)						
<25	ref			ref		
25–44	0.76	0.19	2.99	0.62	0.43	0.89
45–64	0.72	0.17	3.03	0.65	0.44	0.94
65 or more	0.99	0.15	6.35	0.81	0.50	1.31
Facility						
CDCs	ref			ref		
General hospitals	3.36	0.92	12.20	0.82	0.58	1.15
Type of case						
New	ref			ref		
Retreatment	1.49	0.47	4.72	1.49	1.06	2.08
Sputum smear AFB						
Negative	ref			ref		
Positive	0.99	0.32	3.09	1.78	1.27	2.49
Not done/ Unknown	0.89	0.19	4.12	1.21	0.85	1.72
Insurance						
No	0.87	0.31	2.49	1.16	0.86	1.57
Yes	ref			ref		
Level of clinicians						
Junior	0.81	0.31	2.13	0.88	0.68	1.14
Senior	ref			ref		
Site of disease						
Extrapulmonary	0.73	0.15	3.52	0.74	0.45	1.20
Pulmonary TB	ref			ref		

* Adequate dosage (4–6 mg/kg) as the base for comparison. Too high, >6 mg/kg. Too low, <4 mg/kg. RR, risk ratio. CI, confidence interval. CDC, Center of Disease Control (including Tuberculosis Clinical Centre)

**Table 8:** Factors associated with inadequate dosing of rifampicin, Yunnan.

	Lower-than-recommended dosage*			Higher-than-recommended dosage*		
	Relative RR	95% CI Low	95% CI High	Relative RR	95% CI Low	95% CI High
Sex						
Male	1.62	1.14	2.29	0.68	0.37	1.24
Female	ref			ref		
Age groups (years)						
<25	ref			ref		
25–44	1.76	1.01	3.04	0.33	0.15	0.73
45–64	2.79	1.62	4.80	0.28	0.12	0.68
65 or more	1.92	0.95	3.87	1.10	0.47	2.60
Facility						
CDCs	ref			ref		
General hospitals	1.06	0.68	1.66	2.63	0.98	7.06
Type of case						
New	ref			ref		
Retreatment	1.19	0.76	1.85	2.39	1.15	4.98
Sputum smear AFB						
Negative	ref			ref		
Positive	1.07	0.69	1.68	1.17	0.50	2.73
Not done/ Unknown	0.84	0.53	1.31	2.50	1.07	5.88
Insurance						
No	0.78	0.52	1.17	1.11	0.50	2.46
Yes	ref			ref		
Level of clinicians						
Junior	1.17	0.83	1.65	0.67	0.34	1.31
Senior	ref			ref		
Site of disease						
Extrapulmonary	1.13	0.59	2.16	1.93	0.49	7.54
Pulmonary TB	ref			ref		

*Adequate dosage (8–12 mg/kg) as the base for comparison. Too high,>12 mg/kg. Too low, <8 mg/kg. RR, risk ratio. CI, confidence interval. CDC, Center of Disease Control (including Tuberculosis Clinical Centre)

**Table 9:** Factors associated with inadequate dosing of pyrazinamide, Yunnan.

	Lower-than-recommended dosage*			Higher-than-recommended dosage*		
	Relative RR	95% CI Low	95% CI High	Relative RR	95% CI Low	95% CI High
Sex						
Male	1.03	0.69	1.53	0.32	0.24	0.44
Female	ref			ref		
Age groups (years)						
<25	ref			ref		
25–44	0.85	0.49	1.45	0.84	0.55	1.28
45–64	1.01	0.59	1.73	0.65	0.42	1.02
65 or more	2.46	1.29	4.72	0.70	0.36	1.36
Facility						
CDCs	ref			ref		
General hospitals	1.57	0.94	2.62	1.50	0.97	2.31
Type of case						
New	ref			ref		
Retreatment	0.84	0.50	1.38	1.41	0.96	2.06
Sputum smear AFB						
Negative	ref			ref		
Positive	1.64	1.04	2.60	1.13	0.72	1.77
Not done/ Unknown	0.59	0.33	1.06	1.68	1.13	2.49
Insurance						
No	0.62	0.39	0.98	0.95	0.66	1.38
Yes	ref			ref		
Level of clinicians						
Junior	1.57	1.07	2.30	0.94	0.68	1.30
Senior	ref			ref		
Site of disease						
Extrapulmonary	1.18	0.57	2.44	0.71	0.38	1.36
Pulmonary TB	ref			ref		

*Adequate dosage (20–30 mg/kg) as the base for comparison. Too high,>30 mg/kg. Too low, <20 mg/kg. RR, risk ratio. CI, confidence interval. CDC, Center of Disease Control (including Tuberculosis Clinical Centre)

**Table 10:** Factors associated with inadequate dosing of ethambutol, Yunnan.

	Lower-than-recommended dosage*			Higher-than-recommended dosage*		
	Relative RR	95% CI Low	95% CI High	Relative RR	95% CI Low	95% CI High
Sex						
Male	3.52	2.68	4.62	0.51	0.28	0.94
Female	ref			ref		
Age groups (years)						
<25	ref			ref		
25–44	1.31	0.90	1.91	0.46	0.21	1.01
45–64	1.47	1.00	2.17	0.46	0.20	1.04
65 or more	1.35	0.82	2.23	0.79	0.31	2.07
Facility						
CDCs	ref			ref		
General hospitals	0.46	0.32	0.66	1.54	0.61	3.89
Type of case						
New	ref			ref		
Retreatment	0.75	0.54	1.04	2.06	1.05	4.04
Sputum smear AFB						
Negative	ref			ref		
Positive	0.96	0.67	1.38	1.17	0.52	2.66
Not done/ Unknown	0.55	0.39	0.78	1.79	0.77	4.16
Insurance						
No	0.94	0.68	1.28	0.96	0.43	2.12
Yes	ref			ref		
Level of clinicians						
Junior	0.96	0.74	1.26	0.61	0.32	1.16
Senior	ref			ref		
Site of disease						
Extrapulmonary	1.05	0.64	1.75	1.43	0.37	5.56
Pulmonary TB	ref			ref		

*Adequate dosage (15–20 mg/kg) as the base for comparison. Too high,>20 mg/kg. Too low, <15 mg/kg. RR, risk ratio. CI, confidence interval. CDC, Center of Disease Control (including Tuberculosis Clinical Centre)

## Discussion

To our knowledge, this is the largest clinical audit on prescription practice of anti-TB drugs ever conducted in China. A representative sample of more than 2000 new and retreatment TB patients was included in this study. We found a high number of different regimens prescribed for the management of new and retreatment TB; and a high proportion of TB patients without MDR-TB were prescribed second line anti-TB drugs. Furthermore, the dosages of anti-TB drugs prescribed were not consistent with national or international recommendations.

We found that some patients were treated with dipasic rather than INH. Dipasic is a compound formed by chemical combination between isoniazid and p-aminosalicylic acid (PAS) in equimolecular proportions [11–13]. Studies have reported that dipasic is equivalent to INH alone, rather than two drugs (INH and PAS). Patients usually must take 3 tablets of FDC-HP three times per day to obtain sufficient dosages of INH, which is not convenient for most patients. Rifapentine belongs to the rifamycin class of medicine and has a relatively long half-life; it has been recommended for the treatment of latent TB infection, but has not been recommended for routine use in the treatment of active TB. Rifabutin was more expensive than rifampicin and is usually used in patients infected with HIV to reduce the drug-drug interactions with antiretroviral agents. It is not clear why clinicians of the general hospitals in Yunnan used these drugs for a substantial proportion of TB patients.

Our study reveals that regimens used for the treatment of TB were not standardized, with a total of 189 regimens prescribed. The proportion of new TB patients treated with recommended HRZE was 74.8% and that of retreatment patients treated with recommended HRZES was 1.9%, indicating that training and supervision are urgently needed to standardize regimens used in the treatment of TB, especially in general, designated TB hospitals in Yunnan.

A substantial proportion of TB patients were treated with fluoroquinolones, especially retreatment TB patients. This may be detrimental to TB control as irrational use of fluoroquinolones may generate fluoroquinolone resistance. A recent national anti-TB drug resistance survey showed that in China, 5.7% of new TB patients and 25.6% of previously treated TB cases have MDR-TB [2]. Clinicians were more likely to use fluoroquinlones in the treatment of previously treated TB cases. Such practice runs a high risk of generating fluoroquinolone-resistant MDR-TB, which is very difficult to treat [15]. Clinicians in general hospitals were statistically significantly more likely to use fluoroquinolones as compared with those in the CDC TB centers, indicating that it is critical to train clinicians in general hospitals in the rational use of anti-TB drugs. Assessment on the use of fluoroquinolones in the Yunnan TCC shows that the proportion of patients treated with fluoroquinolones decreased significantly, before and after 2013, indicating that training on rational use of floroquinolones was effective in changing prescription practice of anti-TB medicines.

Approximately 10.7% of retreatment TB patients were treated with HRZE plus one second line injectable agent. This deserves attention as second line injectables are recommended for the treatment of MDR-TB and its use in retreatment TB cases prior to the diagnosis of MDR-TB is not recommended. Clinicians in the CDC TB centers were statistically significantly more likely to use second line injectable as compared with general hospitals; this was likely because clinicians in the CDC TB centers were more likely to follow recommendation in using HRZE and an injectable agent (SM) in the treatment of previously treatment cases. However, because of the ongoing shortage of SM, it is likely that clinicians opted instead to use second line injectables. Further assessment is needed to understand why patients without health insurance were more likely to be treated with second line injectables. Assessment on the use of second line injectables in the Yunnan TCC shows that the proportion of patients treated with second line injectables before and after 2013 was not significantly different, also likely due to the shortage of SM.

Assessment of dosages of first line anti-TB drugs prescribed revealed that a substantial proportion of patients were prescribed inadequate dosages of anti-TB medicines. This is of great concern as the use of lower-than-recommended dosages may run the risk of creating drug resistance and the use of higher-than-recommended dosages may increase the risk of adverse drug reactions. The China NTP recommends 300mg INH for all patients, and a substantial proportion of those with low body weight received higher-than-recommended dosages of INH. Whether this practice results in a higher proportion of adverse drug reactions and whether a higher frequency of adverse drug reaction impacted treatment outcomes is not known.

Our study has several strengths. It is a population-based clinical audit with a relatively large sample size using representative samples. Data collection was done by trained health workers using standardized questionnaires; and a broad range of variables on prescription practice was gathered, including both drug type and dosage. Analyses were adjusted for co-variates on the use of fluoroquinolones and second line injectables. The study’s findings allow us to target specific groups to change prescription practice. Our study has weakness. First, we did not assess acquired resistance for fluoroquinolones and second line injectables, which could be obtained in a follow-up study. Second, we did not assess the impact of regimens and dosages on treatment outcome.

Nevertheless, our study sheds light on prescription practice of anti-TB drugs with significant program implication. Findings of irrational use of fluoroquinolones and second line injectables as well as inconsistent dosing of anti-TB drugs should be disseminated and communicated with clinicians. Such findings should be reported in conferences and workshops to fully sensitize clinicians on their potential harmful effects. Training on rational use of anti-TB drugs should be organized, aiming to change prescription practices to be consistent with recommendations. We suspect that the findings of this study may also be generalizable to other provinces in China and would encourage health authorities of other provinces to carry out similar studies.

## Supporting information

S1 Table Recommended dosages of anti-tuberculosis drugs, according to China national tuberculosis programme (by body weight) and WHO (mg/kg). (DOCX)
