# Supplementary material for: Prescription practice of anti-tuberculosis drugs in Yunnan, China: A clinical audit
Source: PLoS One. 2017 Oct 31;12(10):e0187076. doi: 10.1371/journal.pone.0187076 (PMC5663430; doi:10.1371/journal.pone.0187076)
Supplement: S1 Table — (DOCX) [file pone.0187076.s001.docx]

S1 Table . Recommended dosages of anti-tuberculosis drugs, according to China national tuberculosis programme (by body weight) and WHO (mg/kg)

| Name of drugs | China NTP | | WHO |
| --- | --- | --- | --- |
|  | Body weight  <50kg | Body weight  ≥50kg | Dosage in mg/kg (range) |
| Isoniazid | 300 mg | 300 mg | 5 (4–6) |
| Rifampicin | 450 mg | 600 mg | 10 (8–12) |
| Ethambutol | 750 mg | 1000 mg | 15 (15–20) |
| Pyrazinamide | 1500 mg | 1500 mg | 25 (20–30) |

*Dosage in mg per kg body weight.
